# Pattern and Outcome of Traumatic Brain Injury, Addis Ababa, Ethiopia: A Cross-sectional Hospital-based Study

**DOI:** 10.4314/ejhs.v32i2.15

**Published:** 2022-03

**Authors:** Abraham Hagos, Feven Tedla, Abrham Tadele, Ayalew Zewdie

**Affiliations:** 1 Mekelle University, Mekelle, Ethiopia; 2 Department of Emergency Medicine and Critical Care, St Paul's Hospital Millennium Medical College, Addis Ababa, Ethiopia; 3 Department of Neurosurgery, St Paul's Hospital Millennium Medical College, Addis Ababa, Ethiopia

**Keywords:** Traumatic brain injury, emergency, outcome, resource-limited setting

## Abstract

**Background:**

Traumatic brain injury (TBI) is the leading cause of death and disability in young adults in the world. This study assessed clinical characteristics and in-hospital outcomes among traumatic brain injury patients presenting to Addis Ababa Burn, Emergency, and Trauma hospital.

**Methods:**

A cross-sectional hospital-based survey was conducted at AaBET hospital from January 01/2020 to April 30/2020. Data were collected using structured questionnaires from the trauma registry and patient chart. The collected data were analyzed using statistical software SPSS v 25.0.

**Results:**

Among the 304 traumatic brain injury patients, 75% were males with a mean age of 30.4 + 15.7, and 59.2% came from the Oromia region. Road traffic injury was responsible for 45% of the cases, of which pedestrian struck accounts for 52.2% of the cases. Only 50 (16.4%) patients arrived below 02 hours. 201 (66.1%) patients had mild traumatic brain injury the rest had moderate to severe traumatic brain injury. Skullbone fracture (linear, DSF, & BSF) was the most common (n=157, 63.1%) followed by intracerebral lesions (DAI, brain contusion, & ICH) (n=140, 56.5%). Forty-three (14.1%) patients were intubated. 45(14.8%) cases had a neurosurgical intervention. The mortality rate of severe, moderate, & mild TBI were 25%, 8.0% & 2.0% respectively with an overall mortality of 5.6%.

**Conclusion:**

This study showed road traffic injury was the commonest cause of traumatic brain injury which affected young age groups. There was a delayed presentation to AaBET hospital Emergency. The mortality rate was lower than other Ethiopian hospital studies.

## Introduction

Traumatic brain injury (TBI) is the leading cause of death and disability in young adults worldwide, with a devastating impact on patients and their families (1). Nowadays, it has become one of the biggest issues of almost more than 57 million people in the whole world living with the neurological problem rose by TBI, in which 10 million people require hospital-based care (2).

It is a heterogeneous condition in terms of etiology, severity, and outcome. Regarding the etiologies, among all age groups, Road traffic injury (RTI) is the leading cause of TBI in most studies but in some other studies, falling down accident (FDA) comes to be the leading cause and RTI will be the second leading cause of TBI (3–5). TBI affects over 10 million people worldwide each year and is the primary cause of brain injuries and disease (6).

TBI was linked to over 2.5 million ED visits, hospitalizations, or deaths in the United States in 2010, either individually or in tandem with other illnesses, and it led to the deaths of over 50,000 individuals (7). One-third of all head-injured patients in Africa have bad outcomes, and patients with serious head injuries have almost doubled the chance of dying as those in high-income countries (8,9). The incidence of TBI in sub-Saharan Africa, which is estimated to be 150–170/100,000 is much higher than the global incidence, which is estimated to be around 106/100,000 as these countries lack an adequately prepared health system to address the health outcomes associated with TBI (10,11).

Previous research conducted in Ethiopia and other countries of Africa has shown that trauma and, specifically, head injury is a major cause of death and disability (12). However, there was no study from AaBET hospital, since it is new and the first comprehensive trauma center to manage trauma patients in Addis Ababa, Ethiopia. This study assessed clinical characteristics and inhospital outcomes among TBI patients presenting at Addis Ababa Burn, Emergency, and Trauma Hospital.

## Methods and Materials

**Study area, study design and study period**: A hospital-based cross-sectional study of trauma patients who presented to Addis Ababa Burn, Emergency, and Trauma/AaBET/hospital between January 1, 2020, and April 30, 2020.AaBET hospital is part of Saint Paul's Hospital Millennium Medical College which was established in 2015 to improve emergency and trauma care in Addis Ababa, Ethiopia. The hospital has a well-organized emergency department with more than 50 beds divided into red, orange, yellow, and green zones, an intensive care unit (ICU) with 11 beds, including three semi-ICU beds, 130 beds in inpatient departments of orthopedics, neurosurgery, general surgery, and burn unit, and 4 operation theater. In AaBET hospital, more than 30,000 patients are seen per year; out of these, more than 70% are trauma cases. Emergency medical and critical care, orthopedics, neurosurgery, general surgery, and burn care are available 24/7/365. It also offers imaging such as X-rays, ultrasounds, and CT scans, and laboratory facilities. The study period was from January 1, 2020, to September 30, 2020.

**Sample size determination**: Sample size calculated by using the single population proportion formula, prevalence of (0.5) was used where 50 % of patients had TBI from a similar study in Nigeria (15), with a 10% error sample size of 382 patients was included using simple random sampling from a total of 3645 trauma patients seen during the 4 month study period.

**Sampling procedure**: During the study period, patients with TBI from HMIS data were selected, and using simple random sampling 382 charts were assessed. Patients who fulfill the inclusion criteria were analyzed.

**Inclusion and exclusion criteria**: All TBI patients presented to AaBET hospital during the study period were included. Those patients with the wrong diagnosis on patient chart, incomplete data, lost chart, and a dead body on arrival patients were excluded.

## Operational Definitions

**Traumatic brain injury**: any injury to the head associated with any loss of consciousness or confusion/altered sensation and/or documented neuropathology

**Revised Trauma Score (RTS)**: one of the common scores aimed to measure the functional consequences of an injury using three specific parameters (GCS, Systolic blood pressure).

**Multisystem injury**: when there is more than 2 body system injury

**Data collection and analysis**: The data was collected from the emergency room trauma register and the patient's chart using a standardized and pretested data collection sheet. Patient demographics, clinical presentations, and initial diagnosis were collected from the trauma registry while management, outcome and other data which was not available from the trauma registry were collected from the patient chart.

The collected data were entered, cleaned, edited, and analyzed using SPSS 25.0 version statistical software. Descriptive analyses of independent variables were reported as numbers, percentages, and mean ± standard deviation. Binary Logistic regression analysis and multivariate analysis were used to assess the relation of the independent variables with the patient's outcome. Variables with P-value < 0.05 were considered statistically significant. A written legal ethical clearance regarding the study was obtained from St. Paul's Hospital Millennium Medical College IRB. Confidentiality of participants was kept during the study and dissemination of the result.

## Result

**Socio-demographics**: There were 1236 TBI patients out of a total of 3645 trauma patients seen during the study period. This study included 304 patients (78 were excluded), 228 (75%) of whom were males and 76 (25%) of whom were females, with a mean age of 30.41 + 15.68 years, ranging from 2 to 92 years. 214 (70%) patients were in the age group of 16 − 45 years. 180 (59.2%) came from the Oromia region.138 (45.4%) had road traffic injuries, 236 (77.6%) were referred, and 171 (56.1%) came by ambulance (Table 1).

**Table 1:** Socio-demographics of patients with TBI presented at AaBET Hospital, Addis Ababa, Ethiopia, January 01/2020 to April 30/2020

Variables	Classification	Frequency	Percent
	Male	228	75.0
Sex	Female	76	25.0
	< 16	45	14.8
	16–30	142	46.7
Age	31–45	68	22.4
	46–60	32	10.5
	> 60	17	5.6
	Addis Ababa	95	31.3
	Oromia	180	59.2
Region	Amhara	21	6.9
	SNNP	4	1.3
	Other	4	1.3
	RTI	138	45.4
Mechanism of injury	Fall down	54	17.8
	Interpersonal Violence	86	28.3
	Other	26	8.6
Mode of transport	Ambulance	171	56.3
	Contrat Taxi	6	2.0
	Private Car	34	11.2
	Public Transport	79	26.0
	Other	14	4.6
Referral Type	Direct without referral	68	22.4
	Referred without	100	32.9
	communication		
	Referred with	136	44.7

**Clinical features of TBI patients**: At the time of presentation, 63 (20.7%) and 86 (28.3%) patients were triaged to the red and orange areas, respectively, while the rest were triaged to the yellow-green area.201 (66.1%) patients had mild TBI (GCS14-15), the rest had moderate to severe TBI.48 (15.8%) had hypoxia spo2 < 90 and 12 (3.9%) had hypotension SBP < 90 at presentation (Table 2). Fifteen (4.9%) patients had a secondary medical diagnosis, including 7 diabetics, 4 hypertensive patients, 2 cardiac patients, 2 psychiatric patients, and 1 renal patient.

**Table 2:** Clinical profiles of patients with TBI were presented at AaBET Hospital, Addis Ababa, Ethiopia, January 01/2020 to April 30/2020

Variables	Classification	Frequency	Percent
	< 90	12	3.9
SBP	>/= 90	292	96.1
HR	> 100	60	19.7
	60–100	233	76.6
	< 60	11	3.6
SPO2	>/= 90	256	84.2
	< 90	48	15.8
GCS	Severe TBI(3–8)	28	9.2
	Moderate TBI(9–13)	75	24.7
	Mild TBI(14–15)	201	66.1
Triage category	Red	63	20.7
	Orange	86	28.3
	Yellow/green	165	51.0
Pupillary reaction	Normal	266	87.5
	Anisocoric	20	6.6
	Bilateraly Non-reactive	16	5.3
	Difficult to assess	2	.7
Intubation	Yes	43	14.1
	No	261	85.9
Additional medical	Yes	15	4.9
diagnosis	No	289	95.1

Eighty-eight cases (n=88, 28.9%) arrived at AaBET ED from the scene within 02 to 06 hours followed by 70 (23%) greater than 24 hours, 54 (17.8%) 06 to 12 hours, 50 (16.4%) below 02 hours (n=50, 16.4%), and 12 to 24 hours (n=39, 12.8%) from the incident regardless of the mode of arrival.

Two handred and fourty-nine (81.9%) of the total patients had traumatic brain CT findings, but 55 (18.1%) had normal brain CT despite a history of loss of consciousness. Among the brain CT findings, skull bone fracture (linear, DSF, & BSF) was the most common (n=157, 63.1%) followed by Intra-cerebral lesions (DAI, brain contusion, and ICH) (n=140, 56.5%), SDH (n=44, 17.7%), EDH (n=34, 13.7%), and SAH (n=23, 9.2%). Other findings were post-traumatic brain infarction 07 (2.8%), suture diathesis 06 (2.4%), and intraventricular hemorrhage (IVH) 03 (1.2%). One hundred eighty-one (59.5%) patients had two and above brain CT findings (Table 3).

**Table 3:** Brain CT findings with the severity of patients with TBI were presented at AaBET Hospital, Addis Ababa, Ethiopia. January 01/2020 to April 30/2020

**Figure 1:**
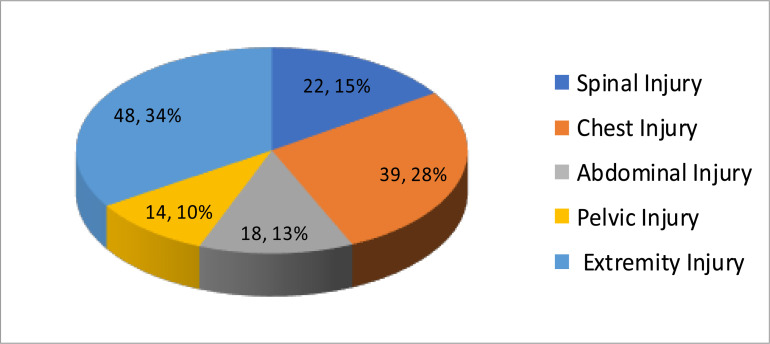
Associated injuries of TBI patients at AaBET ED from Jan 01–Apr 30/2020

Ninety-six (31.6%) of the patients had additional trauma to the other body site, of which 48 (50.0%) had associated extremity injury (Figure 1).

**Management and outcome of TBI patients**: Forty-three (14.1%) patients were intubated; 28 (65.1%) were for airway protection and 15 (34.9%) of them were for the indication of respiratory failure. One hundred sixty-three patients were given phenytoin, 134 patients got TAT, 82 patients received mannitol, and 85 patients received antibiotics.

Sixteen (14.8%) cases had neurosurgical intervention; 27 (60.0%) decompressive craniotomy, 16 (35.6%) DSF elevation, and 05 (11.1%) burr hole with associated duraplasty (22.2%) and cranialization (4.4%). Greater than 46.0% of them had combined more than one type of neurosurgical procedure. Neurosurgical intervention was done for 17 (50.0%) of the patients with EDH, 17 (38.6%) of SDH, 30 (23.6%) of skull fractures, and 04 (22.2%) of those with ICH.

In the ED, 134 (44%) patients stayed for greater than 72 hours before disposition. About 29% of patients were disposed of within 24 to 48 hours, but only 24.7% of patients were disposed of within 24 hours of ED arrival. From the emergency department, 212 (69.7%) were discharged home, 42 (13.8%) were admitted to the neurosurgery ward, 20 (6.6%) were admitted to AaBET ICU, 16 (5.3%) went against medical advice, and 14 (4.6%) of them were referred/linked to other hospitals. One hundred and fourty-five (38.1%) of patients developed at least one complication, of which infection was the most common (n=90, 77.6%) and the remaining are electrolyte imbalance (43, 37.1%), Acute kidney injury (32, 27.6%), and seizure disorders (28, 24.1%). Two hundred fifty four (83.5%) were discharged, 17 (5.6%) transferred/linked to other hospitals, 17 (5.6%) died, 16 (5.3%) patients went against medical advice.

The mortality rate of severe, moderate, & mild TBI were 25%, 8.0% & 2.0% respectively. Binary logistic regression between independent variables and hospital mortality showed age, GCS and co-morbidity had a significant association, while multivariate analysis showed infection and co-morbidity had a strong statistically significant association with a p-value < 0.05 (Table 4).

**Table 4:** Binary logistic regression and multivariate analysis between independent variables and outcome of patient with TBI at AaBET Hospital, Addis Ababa, Ethiopia. January 01/2020 to April 30/2020

Variable	Classification	Alive n=287(94.4%)	Died n=17(5.6%)	COR (CI)	P - Value	AOR (CI)	P - Value
SEX	Male	215(94.3%)	13(76.5%)	0.919(0.290–2.908)	0.885	1.1(1.0–1.1)	0.48
	Female	72(25.1%)	4(23.5%)	1			
AGE	≤35	203(70.7%)	7(41.2%)	1	0.015*	0.16(0.04–0.59)	0.06
	>35	84(29.3%)	10(58.8%)	3.452(1.272–9.373)			
GCS	14–15	197(68.6%)	4(23.5%)	1	0.001*	0.06(0.01–0.39)	0.01
	≤13	90(31.4%)	13(76.5%)	7.114(2.257–22.424)			
Mechanism	RTI	130(45.3%)	8(47.1)	0.932(0.350–2.483)	0.887	0.52(0.11–2.34)	0.32
	NON RTI	157(54.7%)	9(52.9)	1			
Mode of transport	Ambulance	156(54.4%)	15(88.2%)	0.0159(0.036–0.707)	0.016	2.37(0.13–40.5)	0.55
	Non ambulance	131(45.6%)	2(11.8%)	1			
Multisystem injury	Yes	89(31.6%)	7(41.2%)	0.642(0.237–1.747)	0.384	0.96(0.29–3.12)	0.95
	No	198(69.0%)	10(58.8%)	1			
Co-morbidity	Yes	12(4.2%)	3(17.6%)	5.105(1.199–21.734)	0.027*	6.6(0.9–44.8)	0.04*
	No	275(95.8%)	14(82.4%)	1			
Operated	Yes	42(14.6%)	3(17.6%)	0.89(0.222–3.56)	0.869	0.19(0.04–1.05)	0.58
	No	245(85.5%)	14(82.4%)	1			
Complications	Yes	101(35.2%)	15(88.2%)	0.07(0.016–0.32)	0.01	7.5(1.3–37.3)	0.02*
	No	186(64.8%)	2(11.8%)	1			

* P-value <0.05 which is statistically significant

## Discussions

In this study, young aged males were the most common victims of TBI. The major cause of TBI was road traffic injury. These findings were similar to other settings (13,14).

Only Sixty-eight (22.4%) of the cases were presented directly to AaBET ED, which is below that of studies done in Nigeria & Dilla University which were 51.1% and 45% respectively (15,16). This could be because AaBET hospital is a referral trauma hospital for the country. Even though patients were referred from another health facility, only 56.3% were presented to AaBET with Ambulance, which is lower than the study from Brazil Capital (74.0%) (17). Improving prehospital care and referral linkage will improve this challenge.

Only 50 (1.6%) patients came within 2 hours. Studies show mortality will increase with incremental hours of patient arrival (18).

In this study, mild TBI was the most common diagnosis like other settings in Tanzania, England, and Nigeria (3, 15, 19). Skull fracture (51.0%) followed by brain contusion (43.0%), BSF (37.3%) were the common findings that are similar to the studies done in India, Tanzania, Ayder, Dilla university, and TASH (3, 9, 13, 15, 16, 20). Around 31.6% of all the cases had other site injuries which are comparable to the study done in TASH, but lower than that of the study done in central India (50.6%) (9, 13).

Forty-five (14.8%) of the cases had a neurosurgical intervention, which is comparable with the study done in TASH (14.2%), but significantly lower than that of studies done in Ayder (33.5%) and Dilla University (38.2%) (9,16,19). This could be because of the availability of neurosurgeons, 24 hour CT scan, and being a referral center in AaBET and TASH. Hematoma evacuation by either decompressive craniotomy or burr hole was the most common neurosurgical procedure done (55.6%), and most of the patients had moderate TBI at presentation, unlike that of TASH where the number of operated patients was higher in mild TBI (9).

Infection was the most common complication (25.3%) which is associated with higher mortality requires following infection prevention and control procedures in the hospital.

134 (44%) patients stayed greater than 72 hours in the emergency department before disposition, which has a significant impact on the turnover of ED patients and causes ED overcrowding.

The mortality rate in this study was higher than the Saudi Arabian (2.5%) and Nigerian (4.7%) studies, but lower than Ayder (8.3%) & TASH (10.3%) (9,14,15,20). This could be because of the availability of timely CT scans in the ED, early resuscitation in the ED, consultants' decisions, surgical interventions, and nursing care. We suggest having such comprehensive trauma centers in the regions of Ethiopia.

The study has several limitations. Since it was single centered, hospital-based and short study period may not representative of the community.

In conclusion, in this study, RTI was the most common cause of TBI in young people. There was a delayed presentation to AaBET hospital Emergency. The mortality rate was lower than other Ethiopian hospital studies.
